# Ophthalmological Complications of Aesthetic Medicine Procedures: A Narrative Review

**DOI:** 10.3390/jcm14155399

**Published:** 2025-07-31

**Authors:** Lucía De-Pablo-Gómez-de-Liaño, Fernando Ly-Yang, Bárbara Burgos-Blasco, José Ignacio Fernández-Vigo

**Affiliations:** 1 Department of Ophthalmology, Hospital Universitario 12 de Octubre, 28041 Madrid, Spain; depablo.lucia@gmail.com; 2 Department of Immunology, Ophthalmology and ENT, Faculty of Optics, Complutense University of Madrid, 28037 Madrid, Spain; 3 Centro Internacional de Oftalmología Avanzada, 28010 Madrid, Spain; 4 Department of Ophthalmology, Epsom and St Helier NHS University Hospitals, Sutton SM2 5NF, UK; fernandolyyang@gmail.com; 5 Department of Ophthalmology, Hospital Clínico San Carlos, San Carlos Health Research Institute (IdISSC), 28040 Madrid, Spain; bburgos171@hotmail.com; 6 Department of Immunology, Ophthalmology and ENT, Faculty of Medicine, Complutense University of Madrid, 28040 Madrid, Spain

**Keywords:** ophthalmological complications, aesthetic medicine, cosmetic procedures, dermal fillers, botulinum toxin injections, retinal vascular occlusion

## Abstract

Minimally invasive cosmetic procedures, such as dermal fillers, botulinum toxin injections, autologous fat grafting, intense pulsed light (IPL) treatments, and platelet-rich plasma (PRP) treatments, are increasingly popular worldwide due to their convenience and aesthetic benefits. While generally considered safe, these procedures can result in rare but serious ophthalmological complications. The most catastrophic adverse events include central retinal artery occlusion and ischemic optic neuropathy, which may lead to irreversible vision loss. Other complications include diplopia, ptosis, dry eye, and orbital cellulitis, with varying degrees of severity and reversibility. Awareness of potential ocular risks, appropriate patient selection, and adherence to safe injection techniques are crucial for preventing complications. This narrative review summarizes the incidence, mechanisms, clinical features, risk factors, diagnostic approaches, and management strategies of ocular complications associated with aesthetic medical procedures. A narrative literature review was conducted, emphasizing data from clinical studies, case series, and expert consensus published between 2015 and 2025. Special attention is given to anatomical danger zones, the pathophysiological pathways of filler embolization, and the roles of hyaluronidase and hyperbaric oxygen therapy in acute management. Although many complications are self-limited or reversible, prompt recognition and intervention are critical to prevent permanent sequelae. The increasing prevalence of these procedures demands enhanced education, informed consent, and interdisciplinary collaboration between aesthetic providers and ophthalmologists.

## 1. Introduction

Minimally invasive cosmetic procedures have grown exponentially in the past decade. Soft tissue fillers, botulinum toxin injections, autologous fat grafting, and other office-based treatments are now performed worldwide in millions of patients annually [1,2]. In 2014 alone, over 5.5 million filler injections were administered globally, generating more than USD 11 billion in revenue [3]. Hyaluronic acid (HA) fillers are the most commonly used, given their favorable safety profile and reversibility, although collagen inducers, such as calcium hydroxylapatite, poly-L-lactic acid, polymethylmethacrylate (PMMA), and autologous fat transfer procedures, are also employed for volume restoration. Likewise, botulinum toxin type A has become a mainstay for facial rejuvenation, with well over 7 million cosmetic BoNT-A treatments annually in the United States alone (per 2020 ASPS statistics) [1,2]. In addition, energy-based devices such as intense pulsed light (IPL) systems—originally developed for cosmetic indications like hair removal and treatment of vascular and pigmented lesions—have been increasingly adopted in ophthalmology for conditions such as evaporative dry eye (especially due to meibomian gland dysfunction), ocular rosacea, and even in improving ocular surface disease associated with glaucoma therapies. Recognizing infrequent but serious ophthalmological complications is increasingly important with the rising number of procedures [4].

Ophthalmic complications from aesthetic procedures encompass a spectrum ranging from mild, transient effects (e.g., eyelid ptosis or dry eye after botulinum toxin) to permanent blindness from filler embolization [5,6,7,8,9,10,11,12]. Retinal artery occlusion (RAO) caused by inadvertent intravascular filler injection is the most catastrophic event, often resulting in irreversible vision loss [3,13,14,15,16]. The first clusters of filler-induced blindness were reported in the early 2010s (many in East Asia), and by 2015, a literature review had already compiled 98 cases worldwide [9]. Since then, dozens of additional cases have been published, indicating that while the absolute risk is very low, the growing volume of procedures means ophthalmologists and aesthetic practitioners will inevitably encounter such complications [17,18,19,20]. Other important ocular sequelae include oculomotor nerve palsies (e.g., diplopia from toxin diffusion or orbital ischemia), optic neuropathy, periocular tissue necrosis with exposure keratopathy, and ocular surface injuries [12,21,22,23,24].

Key risk factors have been identified, especially for filler injections: certain facial “danger zones” have direct arterial connections to the ophthalmic circulation, notably the glabellar region supplied by the supratrochlear artery, the dorsal nasal artery, the forehead supraorbital area, and the territory of the angular artery near the nose or medial canthus [4,9,17,18,19]. Injections in these areas carry a higher risk of retrograde embolization of filler into the ophthalmic artery, especially if performed with high pressure or improper technique [3,15,16]. Additionally, autologous fat transfer procedures, particularly for treating sunken dark circles, have been linked to a disproportionate number of blindness cases compared to their frequency [7]. This likely reflects fat’s larger droplet size, tendency to cause complete arterial occlusion, and the absence of an enzyme (like hyaluronidase) to dissolve fat emboli.

From an ethical and medicolegal standpoint, these ophthalmic injuries raise concerns. Patients generally do not seek cosmetic treatment expecting to jeopardize their vision; thus, thorough informed consent and disclosure of even rare risks (such as blindness, diplopia, or orbital complications) are recommended by experts and insurers [7]. In many published blindness cases, the treating providers were not ophthalmologists, which can lead to delayed recognition and management. Consequently, consensus guidelines emphasize that any practitioner performing periocular injections must be prepared to identify and manage ocular complications emergently, including having referral pathways to ophthalmology care [4]. This review aims to provide clinicians with a comprehensive understanding of the incidence, mechanisms, clinical features, and evidence-based management of ophthalmologic complications associated with popular aesthetic medicine procedures.

## 2. Methods

### 2.1. Search Strategy and Selection Criteria

We conducted a comprehensive literature search to identify all relevant studies on ocular and periocular complications associated with aesthetic medicine procedures. The primary databases queried were PubMed/MEDLINE, Embase, and Cochrane Library, with supplemental searches in Google Scholar and specific journal archives (e.g., *Ophthalmology*, *JAMA Ophthalmology*, *Dermatologic Surgery*, *Aesthetic Surgery Journal*). The search was primarily focused on studies published between 1 January 2015 and 30 May 2025, in order to capture contemporary practices and data, and was limited to human studies. However, a small number of relevant references outside this range were included when considered essential for contextual or historical purposes. No review protocol was registered for this study. Core search terms included combinations of: “dermal filler”, “botulinum toxin”, “fat graft” or “fat injection”, “intense pulsed light”, “platelet-rich plasma”, “threads”, each paired with terms for ocular complications (“blindness”, “vision loss”, “retinal artery occlusion”, “ptosis”, “diplopia”, “dry eye”, “ophthalmoplegia”, “orbital”, “granuloma”, “cellulitis”, etc.). Reference lists of key articles and prior reviews were hand-searched for additional reports not captured by database queries. A detailed search strategy for each database is provided (see Supplementary Table S1).

Study Selection and Evaluation: To ensure the relevance and quality of the included literature, all titles and abstracts were initially reviewed by two authors. Full-text evaluation was performed on studies deemed potentially eligible. While this process was not structured according to PRISMA standards, we prioritized rigorous judgment in selecting the most clinically meaningful publications. Rather than applying a formal risk-of-bias tool, we qualitatively considered the limitations of each study—particularly the predominance of uncontrolled case reports and small case series (Level III–IV evidence). Potential biases, such as publication bias favoring the reporting of complications, were acknowledged in our narrative synthesis where appropriate.

Inclusion criteria were: (1) peer-reviewed publications (including case reports/series, observational studies, randomized trials, systematic reviews, meta-analyses, and consensus guidelines) that reported or analyzed ocular or periocular complications resulting from cosmetic injectable or minimally invasive procedures; (2) articles focused on human patients; (3) for general reviews or guidelines, the content needed to substantially address ophthalmic outcomes of aesthetic procedures. We included both open-access and paywalled articles (via institutional access) to ensure a comprehensive review of the literature. Non-peer-reviewed sources (e.g., conference abstracts without full text, magazine articles, manufacturer brochures) were excluded. Some large surveys and database studies were included for incidence data and context, even if ophthalmic complications were very rare outcomes.

### 2.2. Data Extraction and Synthesis

From each selected study, we extracted details on the type of cosmetic procedure, patient population, and the ophthalmologic complications observed (including clinical presentation, severity, time to onset, diagnostic evaluation, and outcomes). Specific attention was paid to: vascular complications (e.g., retinal or choroidal artery occlusion, ocular ischemic syndrome), ocular motor nerve or muscle dysfunction (ptosis, diplopia, strabismus), optic neuropathy, ocular surface complications (dry eye, exposure keratopathy), periocular tissue reactions (edema, hematoma, skin necrosis, granuloma, infection), and any reported management strategies or outcomes for these events. When available, incidence or rate data were recorded (e.g., the percentage of cases with a given complication or the estimated risk per number of injections). We also identified risk factors (anatomical locations, injection techniques, filler characteristics) and any preventive recommendations or guidelines.

Given that high-level evidence (e.g., controlled trials) is not available for these rare complications, data were synthesized narratively, triangulating findings from case series, large surveys, and expert consensus documents. The level of evidence for most included publications corresponds to case reports (Level IV) or case series/expert opinion (Level III), as noted in a recent assessment by the American Academy of Ophthalmology. We therefore present an integrative summary highlighting areas of consensus as well as discrepancies. Where possible, numerical data (e.g., number of cases, percentage with improvement) is given to convey the scope of findings. This review is organized by complication category (vascular, motor, ocular surface, etc.) in alignment with the key outcome domains of interest.

To inform this comprehensive narrative review, we performed an extensive literature search using major medical databases. Over 150 publications were reviewed in detail, including systematic reviews, consensus guidelines, larger case series, and individual case reports that document ophthalmological complications of aesthetic procedures. The findings are synthesized below by complication type, emphasizing incidence, clinical presentation, treatment approaches, and prognosis. When available, data from larger cohort studies are included to provide context.

## 3. Results

This review compiles data from a wide range of publications on ophthalmological complications related to aesthetic procedures. The included literature includes systematic reviews, expert consensus documents, observational cohort studies, and numerous single-case reports and small series. For each complication category, we highlight the most relevant findings regarding frequency (when reported), clinical features, treatments, and patient outcomes. Larger case series and high-impact reports are emphasized to give context and support the main clinical insights.

### 3.1. Vascular Complications: Retinal and Ophthalmic Artery Occlusion

Incidence and Mechanism: Intravascular injection leading to retinal artery occlusion (RAO) or ophthalmic artery occlusion (OAO) is the most feared complication of dermal fillers and autologous fat transfer. Fortunately, it is exceedingly rare, estimated to be 1 in 100,000 filler injections [4].

As of 2019, a global literature review had identified 98 cases of filler-induced blindness up to 2015 and an additional 48 cases from 2015 to 2018 [9]. A recent Ophthalmic Technology Assessment by the American Academy of Ophthalmology (AAO) in 2025 compiled reported cases and found 198 patients with vision loss caused by cosmetic filler emboli [7]. Of these, 83% were attributed to hyaluronic acid (HA) fillers, 15% to autologous fat, and the remainder to collagen, calcium hydroxylapatite, or poly-L-lactic acid [7]. The anatomical sites most often associated with filler-induced RAO/OAO are the nasal region (up to ~40–56% of cases), followed by the glabellar forehead region (~25–27%), then the frontal scalp/brow (≈7–19%) and nasolabial folds (~4–15%) [7,9]. This distribution reflects the presence of arteries in these areas (dorsal nasal, supratrochlear, supraorbital, and angular arteries) that directly or indirectly communicate with the ophthalmic artery circulation of the eye [3]. These are often referred to as “danger zones” due to their arterial connections to the eye.

The underlying pathophysiology involves accidental filler injection into an arterial vessel, with retrograde material flow under high injection pressure into the ophthalmic arterial tree, followed by anterograde embolization to the retinal or choroidal circulation when the pressure is released [4,15,16]. In vitro studies indicate that injection pressures > 150–300 mmHg (easily achieved with forceful plunger depression) can overcome arterial blood pressure and drive filler proximally [4]. Given the small luminal caliber of the ophthalmic branches, as little as 0.05–0.1 mL of filler may be sufficient to occlude critical arteries. Once in the ophthalmic artery, the embolus typically lodges in the central retinal artery (CRA) or posterior ciliary arteries, causing instant cessation of blood flow to the retina and optic nerve [25,26]. Frequently, both retinal and choroidal circulations are compromised, as evidenced by combined CRAO and ophthalmic artery occlusion in many cases. Larger filler particles, such as autologous fat, are more likely to cause complete arterial occlusion than smaller, more deformable materials like HA. This catastrophic sequence highlights the importance of appropriate injection techniques, knowledge of facial vascular anatomy, and constant vigilance to prevent intravascular injection. Risk factors include injection into high-risk anatomical zones (e.g., glabella, nasal dorsum), use of sharp needles instead of blunt cannulas, forceful injection techniques, and filler materials with larger particle sizes such as autologous fat. These factors increase the likelihood of retrograde embolization and subsequent vision-threatening complications.

Clinical Presentation: The onset of vision changes is immediate in virtually all reported cases of filler-induced occlusion. Patients often experience sudden, profound vision loss in one eye, classically noted as loss of vision during injection or seconds thereafter, sometimes accompanied by severe eye pain [7,25].

Associated symptoms can include ophthalmoplegia (limited eye movements) and ptosis if the orbital circulation (ophthalmic artery) is occluded proximally, affecting the extraocular muscles and eyelid (via ischemia of the superior division of CN III or the muscles themselves) [9]. Patients and injectors may also notice an acute blanching or discoloration of the skin in the injected area (e.g., glabellar blanching or livedo), indicating tissue ischemia; skin necrosis occurs in roughly 44% of blindness cases [9]. Frequently, patients report other transient neurologic symptoms such as headache, dizziness, nausea, or even loss of consciousness, which in some cases can signal concomitant cerebral embolization leading to stroke. Indeed, filler emboli can pass into the internal carotid artery and cause ischemic stroke in the anterior or middle cerebral artery territories; about 12–19% of cases of filler blindness have accompanying cerebral infarcts on imaging [3,7].

Occlusion of the CRA appears as inner retinal infarction, presenting as a pale retina with a cherry-red spot. There may be segmented (“boxcar”) blood flow in the retinal arterioles. While occlusion of posterior ciliary branches can lead to choroidal ischemia and infarction of the optic nerve head, known as anterior ischemic optic neuropathy. Initially, the retina may look relatively normal with early optic disk pallor, or there could be combined signs of retinal and choroidal ischemia [25]. Fluorescein angiography shows delayed or absent filling of the retinal artery and patchy choroidal non-perfusion. Over days to weeks, the optic disk turns pale and becomes atrophic, and the retinal inner layers undergo atrophy. Some cases later develop neovascular complications or exudative retinal detachments due to ischemia-triggered release of Vascular Endothelial Growth Factor (VEGF).

Unfortunately, the prognosis for vision after filler-induced retinal/ophthalmic artery occlusion is poor. In the AAO 2025 review of 198 cases, final visual acuity was no light perception (NLP) in ~70% of eyes [7]. Only ~28% of patients had any improvement in vision after the event, and even fewer (≈1 in 5) attained a functionally meaningful recovery (e.g., reading vision). A minority of cases have retained some peripheral or partial central vision (e.g., counting fingers), often when the occlusion was incomplete or a branch retinal artery rather than the central trunk was embolized [7,9]. There are isolated reports of nearly complete vision recovery—for example, one case of HA filler CRAO treated within an hour with aggressive measures regained 20/20 vision [27]. However, such outcomes are exceptional. Visual field defects and cranial nerve palsies (if present) may partially recover if due to transient ischemia, but optic atrophy typically ensues in total occlusions. Notably, autologous fat emboli have been associated with particularly severe outcomes (often NLP vision with extensive stroke), whereas small-particle HA filler might, in theory, lodge more distally, occasionally sparing some vision [3,7]. Overall, the data show no significant difference in prognosis by filler type—both HA and fat emboli yielded devastating, primarily vision loss. Bilateral blindness is extremely rare but has been recorded in a few cases when emboli likely entered the internal carotid artery, affecting both ophthalmic arteries [7].

There is no proven effective treatment for filler-induced retinal artery occlusion; however, various interventions have been attempted in the acute setting. Standard CRAO emergency therapies (ocular massage, anterior chamber paracentesis to lower intraocular pressure, carbogen inhalation) are often tried first. Additionally, since HA is degradable, practitioners often administer hyaluronidase, an enzyme that breaks down HA filler. Various methods have been used: intraarterial hyaluronidase via catheterization of the ophthalmic artery, retrobulbar (orbital) injections of hyaluronidase, and subcutaneous injections around the eyes and forehead [4,7,28]. There are numerous case reports, but the evidence on whether hyaluronidase meaningfully improves outcomes is mixed; a 2019 systematic review concluded there was insufficient evidence that retrobulbar hyaluronidase alters the clinical course [4]. It may help dissolve HA in the local circulation, but often by the time it is administered (usually more than 1 h after occlusion), irreversible retinal damage has already occurred. Nonetheless, the current consensus is that if HA filler is suspected, high-dose hyaluronidase (450–1500 units) should be administered via retrobulbar injection as soon as possible, since the potential benefit outweighs the minimal risk [4,7]. Some experts recommend repeating orbital hyaluronidase injections over 24–48 h. Intra-arterial fibrinolysis with tissue plasminogen activator (tPA) directly into the ophthalmic artery has been attempted in multiple cases, especially in Korea; while some cases have shown partial vision recovery, there is also significant risk (such as intracerebral hemorrhage) and no consistent improvement across cases [3,7]. The AAO report found that among cases receiving intra-arterial treatments (hyaluronidase or tPA), outcomes were not clearly better than those managed supportively [7].

Adjunctive therapies include systemic corticosteroids to reduce inflammation and secondary swelling, IV mannitol or acetazolamide to lower intraocular pressure and potentially improve perfusion pressure, and hyperbaric oxygen therapy (HBOT). HBOT can enhance oxygen delivery to the ischemic retina via the choroidal circulation and has shown anecdotal benefits in some reports—for example, a case of CRAO with hand motion vision improved to 20/40 after a course of HBOT started within 1–2 days [4,7,27]. However, most cases begin HBOT late (days after occlusion) and see little change. Surgical decompression, such as pars plana vitrectomy with inner retinal massage, has been attempted to dislodge emboli but with no proven success. In summary, no treatment reliably reverses filler-induced vision loss [4]. Prevention remains essential. Once filler embolization occurs, the focus should be on timely recognition, immediately stopping injection, and starting the above measures as early as possible—ideally within the first 60–90 min, which is generally considered the window before retinal infarction becomes irreversible. In practice, however, most patients do not regain useful vision even with maximal therapy. Visual rehabilitation with low-vision aids and counseling is then recommended.

Regarding the specific risk factors that increase the risk of complications, the anatomic danger zones for filler injection are clearly outlined by multiple sources [4,9]. The highest-risk areas—the nasal bridge (nasofrontal angle) and the glabellar region—should be treated with extreme caution. Many practitioners avoid sharp needles and opt for microcannulas in these zones, injecting very slowly with small amounts (<0.1 mL per bolus), and aspirating before injection (although the effectiveness of aspiration in preventing intravascular injection is debated, as small needles can become occluded). Using the lowest effective syringe pressure is recommended—one guideline suggests applying pressure with the thumb on the plunger (which produces lower pressure than the index finger) [4]. Patient factors, such as prior surgery or scarring, can alter vessel pathways, but it remains unclear whether they increase risk or sometimes provide protection by blocking vessels. Practitioner training and technique are key modifiable factors—many cases of blindness have happened when medical professionals were not formally trained in oculofacial anatomy (e.g., unlicensed providers or those performing off-label injections) [3]. Therefore, expert consensus strongly supports specialized training, credentialing, and informed consent when injecting in high-risk facial areas. Being prepared with a complication kit (including hyaluronidase, aspirin, etc.) and an emergency referral plan to an ophthalmologist can ensure prompt action if an occlusion occurs. Table 1 summarizes essential clinical and preventive insights regarding ophthalmologic complications associated with aesthetic medicine procedures.

It should be noted that platelet-rich plasma (PRP) injections using autologous fluid have also been reported to cause retinal artery occlusion via the same mechanism. At least seven cases of blindness from cosmetic PRP facial injections have been published, including a series of four patients injected in the glabella or nasolabial fold by cosmetologists [25]. PRP is liquid, but it can still act as an embolic agent (possibly by thrombus formation or platelet aggregation). No antidote exists; management is similar to that of filler emboli. The occurrence of RAO with PRP underscores that any injectable material placed in the wrong location can occlude critical ocular vessels.

In summary, filler and fat injections can unpredictably cause retinal or ocular ischemia. While rare, the severity of this complication warrants vigilance. Table 2 outlines the most significant ophthalmologic complications associated with aesthetic medicine, including their estimated frequency, management strategies, and relevant clinical notes.

### 3.2. Ocular Motility Disorders: Ptosis, Diplopia, and Strabismus

Eyelid Ptosis (Blepharoptosis): Transient drooping of the upper eyelid is a common complication of botulinum toxin A (BoNT-A) injections in the upper face. Diffusion or misplacement of BoNT-A can weaken the levator palpebrae superioris or Müller’s muscle, leading to unilateral upper eyelid droop. In clinical trials of cosmetic onabotulinumtoxinA for glabellar lines, upper eyelid ptosis occurred in about 2.5% of treated patients [6,12]. A large systematic review (35 studies, over 8700 patients) also found a blepharoptosis incidence of 2.5% and brow ptosis in 3.1% of patients receiving BoNT-A to the upper face. Ptosis typically begins a few days to a week after injection and can last 2 to 8 weeks (rarely up to 3 months in persistent cases) [31]. Patients may notice a heavy or asymmetrical lid. On examination, there is mild to moderate droop (often 1–3 mm) and decreased margin reflex distance; extraocular movements are normal, helping to distinguish it from a CN III palsy. Ptosis from BoNT is dose-dependent and linked to injector technique—it occurs most often with glabellar (frown line) injections if toxin diffuses through the orbital septum to the levator muscle. To reduce this risk, injectors should maintain a distance of more than 1 cm above the superior orbital rim in the glabella and forehead, use the lowest effective dose, and avoid massage or downward pressure immediately after injection.

Diplopia (Double Vision): Cosmetic botulinum toxin around the eyes can also cause paresis of the extraocular muscles, leading to diplopia. This is much rarer than ptosis, but a review of case reports suggests an incidence of about 0.7–2.2% in cosmetic patients [21,23]. Common scenarios include weakness of the lateral rectus muscle after toxin injections to the crow’s feet (lateral canthal region) and weakness of the inferior or superior oblique muscles after injections around the medial lower eyelid or brow [21]. Patients may develop horizontal or vertical diplopia, sometimes with an abnormal head posture to compensate (e.g., head tilt in superior oblique palsy) [21]. Onset usually occurs within the first days to one week after injection, and as with ptosis, the issue is temporary—most cases resolve within 4–6 weeks as the botulinum toxin’s effects wear off [22]. Transient diplopia following botulinum toxin-induced strabismus, such as esotropia secondary to medial rectus overaction, has been reported to resolve spontaneously within few months in many cases [29]. Management typically involves conservative measures: prism glasses or an eye patch can alleviate symptoms while waiting for recovery. There have been explorations of using antitoxin or botulinum injections into the antagonist muscle to counteract the effect (e.g., injecting botulinum toxin into the medial rectus to balance a lateral rectus palsy). One case report described injecting a small dose of botulinum toxin into the contralateral eye muscle to treat persistent diplopia, which corrected the deviation until the original toxin wore off [30]. However, such approaches are used sparingly. Apraclonidine eyedrops (which stimulate Müller’s muscle) are effective for ptosis but do not address ocular misalignment; they are primarily used for ptosis (see below).

It is important to note that filler injections can also cause ophthalmoplegia, but through a different mechanism—ischemia of cranial nerves or extraocular muscles within the orbit. In cases of filler-induced OAO, concurrent palsies of the third, fourth, or sixth cranial nerves have been observed, presenting as ophthalmoplegia and diplopia alongside vision loss [9]. These ischemic cranial neuropathies tend to be permanent or long-lasting due to nerve infarction and generally have a worse prognosis than toxin-induced palsies. For instance, if filler blocks the blood supply to the lateral rectus muscle or abducens nerve, it can lead to sixth nerve palsy with esotropia, which often does not fully recover. In the AAO filler blindness series, ophthalmoplegia was noted in a significant portion of cases (around 19%) [7]. Typically, this occurred in instances of ophthalmic artery occlusion where orbital tissues suffered infarction. Management in these cases follows stroke protocols, such as administering steroids for nerve inflammation, and later considering strabismus surgery or prism correction if misalignment persists.

Brow Ptosis: Although not a direct ocular health issue, brow ptosis (descent of the eyebrow) caused by botulinum toxin can contribute to a sensation of a “heavy” upper eyelid and relative pseudoptosis. The incidence is similar to eyelid ptosis (~3% in clinical studies) [6]. Severe brow ptosis can narrow the visual field superiorly. Treatment typically involves watchful waiting; in some cases, strategic placement of additional botulinum toxin in the brow depressor muscles (orbicularis oculi) can paradoxically lift a drooped brow by allowing the frontalis muscle to act unopposed. Patients are advised beforehand about this possibility.

Management of Ptosis: The primary treatment for BoNT-induced eyelid ptosis is topical apraclonidine 0.5% or 1% drops, an α-adrenergic agonist that stimulates Müller’s muscle to lift the upper eyelid by 1–3 mm within minutes of application [31]. Patients use it two or three times daily until the ptosis resolves. It does not correct large ptosis (>3 mm) but can help improve symmetry. If apraclonidine is ineffective or contraindicated, simply observe; toxin effects typically subside within 4–6 weeks. For persistent ptosis lasting longer than 3 months (which is very rare and may be due to antibody formation or coexisting issues), referral to oculoplastics can be considered, although surgical intervention is almost always unnecessary for iatrogenic ptosis caused by botulinum toxin [31]. Importantly, patients with new-onset ptosis after cosmetic injections should be evaluated for signs of ocular ischemia or neurologic problems (such as pupil reactions and eye movements) to confirm it is truly a benign toxin effect.

In summary, botulinum toxin complications affecting ocular motility are temporary and manageable. Using proper injection techniques (such as precise injection points, correct dosing, and avoiding orbital septum penetration) and selecting suitable patients help keep their occurrence low. However, providers should inform patients of these risks, as even brief drooping or double vision can cause discomfort and functional issues (like difficulty driving) until they resolve [21].

### 3.3. Ocular Surface and Adnexal Complications: Dry Eye, Exposure Keratopathy, and Soft Tissue Reactions

Dry Eye and Exposure Keratitis: Cosmetic procedures around the eyes can disrupt eyelid function and tear film, causing ocular surface discomfort. The most common cause is botulinum toxin-induced orbicularis oculi weakness, which can impair blinking and eyelid closure. After lateral canthal BoNT injections (for crow’s feet), patients may experience dryness, burning, or tearing due to incomplete blinking and decreased tear distribution [6,31]. In the systematic review by Cavallini et al., about 3% of patients had “eye sensory disorders” (dryness, irritation, or blurred vision) after cosmetic BoNT injections to the upper face [6]. Another study reported lagophthalmos (inability to fully close the eyelids) in a small subset of patients following lower eyelid botulinum toxin, which can lead to nocturnal corneal exposure [32]. These effects are dose-dependent and usually mild. Management includes using lubricating drops and ointment at night until muscle function recovers. If significant exposure keratopathy (corneal staining) occurs, patients should apply aggressive lubrication, use moisture goggles during sleep, and possibly tape their eyelids shut at night. Fortunately, as orbicularis tone returns (in 2–3 months), normal blinking resumes and dry eye symptoms improve. For severe dryness during this period, temporary punctal plug insertion may be considered. In rare cases where eyelid closure is severely impaired, a temporary partial tarsorrhaphy (suturing the outer canthus) can protect the cornea, although this is very uncommon in cosmetic procedures.

Epiphora (Tearing): Paradoxically, some patients experience tearing after botulinum toxin—this can be reflex tearing from dry eye or, occasionally, from lower lid laxity causing poor tear drainage. Lower lid botulinum toxin (for wrinkles or “jelly roll”) can weaken the pretarsal orbicularis muscle that pumps tears into the lacrimal canaliculi, leading to tearing. This effect is temporary and improves as muscle tone returns. No specific treatment is necessary beyond managing any dryness [32].

Conjunctival Injection and Edema: Any periocular injection (filler, PRP, mesotherapy, etc.) carries a risk of localized swelling, bruising, and a mild inflammatory response. It is common for patients to have a red eye (subconjunctival hemorrhage or reactive conjunctival injection) after injections near the eye—for example, needle entry through the eyelid can cause a small subconjunctival bleed. This redness usually resolves within 1–2 weeks. If significant chemosis (conjunctival edema) occurs due to eyelid inflammation or allergic reaction, topical decongestants or steroids can be used, but this is rarely necessary [33,35].

Periorbital Edema and Ecchymosis: Bruising around the eyelids is a common minor complication of any injectable due to the region’s vascularity. It occurs in a significant number of patients—for example, one study on tear trough fillers reported mild ecchymosis in approximately 19% of treatments, all of which resolved on their own. Periorbital edema can result from the trauma of injection or from the hydrophilic nature of HA fillers drawing fluid into the tissue. Most edema is mild and resolves within days. However, persistent swelling can happen after tear trough filler if the product is placed too superficially or in excessive volumes; it appears as prolonged puffiness of the lower lids. This is often caused by the Tyndall effect (bluish fluid retention) or lymphatic blockage. Treatment involves injecting hyaluronidase to dissolve the filler, which typically leads to resolution of the swelling within a week or two. Delayed-onset unilateral swelling months after filler may indicate an inflammatory nodule or localized infection (see below). Patients should sleep with their head elevated and use cold compresses after injections to reduce typical swelling [36].

In thread lift procedures, edema and bruising are also the most common immediate issues—one study from China found 40.5% of patients had some cheek dimpling or swelling after thread placement. These were mostly mild and resolved with massage or spontaneously. If a thread causes a localized hematoma, firm pressure and possibly needle aspiration of the clot may be used. Thread placement in the brow or midface can sometimes worsen eyelid edema if lymphatics are disrupted, but serious or lasting edema is rare with modern PDO threads [37].

Skin Necrosis and Scarring: Although not an intraocular complication, skin necrosis in the periorbital area can indirectly affect the eye (e.g., leading to exposure). Filler injection can cause skin ischemia if arterial supply to the skin is occluded (for example, necrosis of the glabella or bridge of nose). Periocular skin necrosis (e.g., of the upper eyelid or bridge) has been reported and can lead to scarring that subsequently causes eyelid retraction or poor closure. In ophthalmic complications, skin necrosis occurred in about 44% of filler-induced blindness cases [9], often in the glabellar region, and in some PRP-induced cases, resulting in scars on the glabella and forehead [25]. Managing impending skin necrosis involves hyaluronidase injections to restore blood flow if caused by HA filler, along with warm compresses, nitroglycerin paste, and hyperbaric oxygen as additional treatments to improve circulation. Any areas of necrosis are treated with topical antibiotics and wound care to prevent infection. After healing, patients may need scar revision; if eyelid malposition occurs (e.g., an upper eyelid scar causing ptosis or lagophthalmos), oculoplastic surgery may be required. These situations are complex and, thankfully, infrequent.

### 3.4. Delayed Inflammatory Reactions: Granulomas and Nodules

Delayed-onset granulomatous nodules can occur weeks to years after filler implantation due to a foreign-body reaction or low-grade infection (biofilm). These appear as firm, often tender subcutaneous nodules. Around the eyes, this may occur after tear trough filler or brow filler [25]. The incidence of true granulomas is low, around 0.01–0.1% with modern HA fillers. A large five-year retrospective study reported granulomatous or immune-related nodules in less than 1% of patients, mostly with non-HA fillers. Polymethyl methacrylate (PMMA) and silicone have higher granuloma rates—up to several percent—due to their permanence. This review identified multiple case reports of periocular filler granulomas causing chronic swelling or erythema in the eyelids. For example, filler in the tear trough sometimes developed a persistent palpable lump months later, unresponsive to simple massage.

Diagnosis is clinical but can be confirmed by biopsy (showing foreign-body giant cells).

Treatment for HA filler nodules involves hyaluronidase injections, which can dissolve the remaining filler and often improve the reaction. Additionally, intralesional corticosteroids (e.g., triamcinolone 2.5–10 mg/mL) are injected into the nodule to suppress granulomatous inflammation [33,34]. Multiple sessions may be needed. Another option is intralesional 5-fluorouracil (5-FU), often combined with a steroid, which has been reported to help resolve refractory granulomas. If infection (biofilm with organisms such as atypical mycobacteria) is suspected, a course of appropriate antibiotics (e.g., clarithromycin for atypical mycobacteria, or fluoroquinolone and macrolide combinations) is indicated, sometimes for several weeks. Cultures or PCR from aspirated material can guide therapy. In resistant cases or for non-HA fillers, surgical excision of the nodule may be required. Around the eye, care must be taken with excision to avoid damaging orbital structures. Prevention of granulomas centers on using proper injection techniques (aseptic technique to avoid introducing bacteria, placing the filler at the correct depth) and perhaps pre-procedure antibiotics in high-risk patients, though routine antibiotic prophylaxis is not standard.

### 3.5. Angioedema and Allergy

Though extremely rare, immediate hypersensitivity reactions to fillers or botulinum toxin can cause periorbital angioedema [38,39]. There are case reports of rapid eyelid swelling and urticaria after HA filler in patients with prior severe allergies. Treatment is with antihistamines, corticosteroids, and if severe, epinephrine. Most HA fillers are non-animal and have low immunogenicity; hypersensitivity incidence is <0.5% in studies. An allergy to botulinum toxin is even rarer, as it is a purified protein, but some patients can develop antibodies that reduce effectiveness rather than cause allergy. Filler products containing lidocaine could trigger eyelid swelling in those with lidocaine allergy, so a non-lidocaine version should be used for sensitive individuals.

### 3.6. Thread Lift Complications

Threads (e.g., PDO, PLLA, or other sutures) used for facial lifting often cause localized tissue reactions. In a large series of 190 thread lift patients, complications included skin dimpling (40%), contour irregularities (17%), thread visibility (16%), thread extrusion (5%), and infection (9%) [37]. Most of these were minor and managed conservatively, such as massage for dimpling, partial thread removal for visibility, and antibiotics for infection. Notably, granuloma formation can occur if a thread is placed too superficially or if the suture material triggers a reaction. In the periocular region, barbed threads are sometimes used for brow lifts—complications may include palpable subcutaneous knots or, rarely, a thread end poking out near the brow. If a thread migrates or protrudes, it is usually removed. Infections tend to cluster around thread entry or exit points; Staphylococcal abscesses have been observed along the thread path. High-dose antibiotics and thread removal are the primary treatments in these cases.

Importantly, thread lifts have not been reported to cause vision loss or ocular motor palsy—their complications are limited to soft tissue [37]. One theoretical concern is whether a deep temple thread could injure the orbital septum or trochlea of the superior oblique, but no such cases are found in the literature. Therefore, threads are relatively safe for ocular structures, with the warning that severe infection could secondarily spread to the orbit (for example, if untreated, leading to orbital cellulitis, which would present with painful swelling, erythema, and possibly diplopia). We found no documented cases of orbital cellulitis from thread lifts, but any infection in the eyelid or brow region should be monitored and treated aggressively to prevent spread.

### 3.7. Optic Neuropathy and Neurologic Sequelae

Vision loss from cosmetic procedures can also occur due to optic nerve damage, even in the absence of an arterial occlusion of the retina. The most direct cause is ischemic optic neuropathy resulting from filler embolization—for example, occlusion of the posterior ciliary branches of the ophthalmic artery can lead to anterior ischemic optic neuropathy (AION) with optic disk infarction [25]. Clinically, this is hard to distinguish from CRAO in the early stages, as both cause severe vision loss. If choroidal perfusion remains somewhat preserved but the optic nerve becomes ischemic, a patient might experience optic disk swelling and vision loss despite initially intact retinal circulation. However, this scenario is less common, as most filler occlusions affecting the optic nerve also affect the central retinal artery. The end result in either case is optic atrophy and permanent vision loss.

Another mechanism involves optic nerve compression caused by filler leading to an acute compartment syndrome in the orbit. A large-volume filler injected improperly into the orbital space (for example, too deep in the glabellar region penetrating the orbital septum) can cause a retrobulbar hemorrhage or filler mass, resulting in a sudden increase in orbital pressure and optic nerve compression. A case reported in 2022 described orbital compartment syndrome after tear trough hyaluronic acid filler, likely due to retrobulbar vascular injury and hemorrhage; emergency lateral canthotomy and cantholysis were performed, preventing permanent blindness [40]. The patient recovered without visual deficits, making it the first documented case of orbital compartment syndrome caused by filler. This emphasizes that, even without intra-arterial injection, periocular filler can threaten the optic nerve through mechanical means. Clinicians injecting around the orbital rim should watch for signs such as acute proptosis, tense orbit, or vision loss—performing a surgical canthotomy to decompress the orbit within minutes is crucial if orbital pressure compromises the optic nerve. Fortunately, such cases are exceedingly rare.

### 3.8. Cerebral Infarction and Neurologic Deficits

As mentioned earlier, filler emboli can cause ischemic stroke along with ocular effects. Neurologic deficits like hemiparesis, aphasia, or altered mental status may accompany vision loss when filler enters the internal carotid circulation. In Zhao et al., of 12 filler-blind patients studied with MRI, 5 had multifocal acute brain infarcts on diffusion-weighted imaging, mostly in the frontal and parietal lobes [3]. Two patients experienced contralateral hemiplegia and even urinary incontinence from large territory strokes. These strokes are life-threatening; one patient in that series died due to extensive cerebral infarction and hemorrhagic transformation. Therefore, from an ophthalmic perspective, any sudden blindness after facial injection should prompt not only ocular intervention but also neurologic evaluation with brain MRI to assess for stroke. Treatment of such strokes follows standard protocols; IV tPA is contraindicated if filler was the cause, due to uncertain benefit and risk of hemorrhage, but some interventionalists attempt thrombectomy in cases where appropriate. Prognosis for these strokes is variable—small emboli might cause only transient ischemic attacks, whereas large fat emboli can be fatal. This crossover into neurology reinforces the need for multidisciplinary management (ophthalmology, neurology, neuroradiology) in severe filler complications.

### 3.9. Infectious and Immunologic Sequelae

Proper aseptic technique makes infectious complications infrequent, but they do occur. For botulinum toxin, infection risk is negligible (the small needle and bacteriostatic properties of the solution make it very safe). With fillers, especially HA fillers, cases of delayed bacterial infection or biofilm have been reported, typically manifesting as tender nodules or abscesses weeks after injection [34].

Common organisms include *Staphylococcus epidermidis*, *Propionibacterium acnes*, or atypical mycobacteria that form biofilms on filler material. Around the eyes, an infected filler nodule may present as a red, warm swelling in the eyelid or infraorbital area. Acute infections (within a few days) are more likely caused by streptococcal or staphylococcal contamination and can lead to cellulitis [34]. For instance, a case of preseptal cellulitis after tear trough filler might show eyelid erythema, swelling, and mild fever. Treatment is with appropriate antibiotics (e.g., a beta-lactam for streptococcal cellulitis). If an abscess forms, drainage is indicated. Serious deep infections like orbital cellulitis are extremely rare from cosmetic injections, but not impossible if a needle introduces bacteria into the orbit or if a periorbital infection is neglected. No direct literature search results were found in our study linking orbital cellulitis to filler or threads; however, one could conceive a scenario where infected filler in the glabella erodes through tissue planes. Any sign of orbital involvement (proptosis, pain with eye movement, ophthalmoplegia) in the context of facial injection demands urgent evaluation (CT scan) and IV antibiotics to prevent permanent vision loss from infectious optic neuropathy.

Herpetic Reactivation: Periocular injections can reactivate HSV or VZV in patients with a history. A case of herpes zoster ophthalmicus was reported after PRP injections for scalp alopecia, presumably due to an immune trigger [40,41]. While not a direct filler complication, any severe viral reactivation in the trigeminal distribution after the procedure should be recognized and treated with antivirals.

In summary, infectious and immune complications of cosmetic procedures around the eyes are uncommon but important. They usually present as subacute phenomena (occurring days to weeks post-procedure) rather than immediately. A high index of suspicion, along with appropriate cultures/biopsies, helps differentiate sterile inflammatory nodules from infections. Treatment tailored to the cause (antibiotics for infection, steroids for inflammation) generally leads to resolution, though some chronic granulomas may persist and require surgical excision.

## 4. Ocular Complications of Energy-Based Facial Procedures (IPL and Laser)

*Intense Pulsed Light* (*IPL*): IPL devices are commonly used for facial skin rejuvenation, treatment of pigmented lesions, and hair removal. Periocular IPL treatment can result in ocular damage if the eyes are not adequately shielded. The pigmented intraocular structures, especially the iris, absorb light in the wavelength range emitted by IPL. As a result, energy from IPL can induce acute anterior uveitis (inflammation inside the eye) and damage to the iris. For example, Lee et al. reported two cases of severe ocular injury following cosmetic IPL to the face [42]. Both patients developed marked eye pain, pupillary constriction, and anterior uveitis within hours of treatment, progressing to iris atrophy with permanent photophobia due to iris transillumination defects [42]. In one case, posterior synechiae (iris adhesions) formed and the iris pigmentation was partially ablated, leaving the patient with persistent pain and light sensitivity years later [42]. Similarly, Jewsbury and Morgan described a case of uveitis and iris photoablation secondary to IPL therapy, resulting in iris defects and chronic visual disturbances [43]. Additional reports have documented IPL-induced iritis (iridocyclitis); for instance, an IPL treatment for a medial canthal capillary malformation led to acute unilateral iritis in a patient, presumably from light energy penetrating the eye near the nasal canthus [44]. These cases underscore that IPL, though not a laser, can permanently affect intraocular tissues. The common contributing factor was a lack of appropriate eye protection during treatment. Patients often did not wear proper opaque eye shields, allowing IPL flashes to reach the ocular structures. Hence, stringent eye protection (metallic corneal shields or well-fitted goggles) is mandatory for any IPL procedure around the eyes [45]. Treating physicians and cosmetic specialists must be aware that IPL can cause potentially blinding complications and should take precautions accordingly [42].

*Laser Hair Removal* (*Diode*, etc.): Cosmetic laser systems (such as diode lasers at 810 nm or alexandrite lasers ~755 nm) used for hair removal around the brows and eyelids have also caused ophthalmic injuries [45,46,47]. Laser energy can directly penetrate the thin eyelid tissue and be absorbed by ocular structures if adequate shielding is not provided. Cataract formation and iris damage have been reported from periocular laser hair removal. Brilakis and Holland documented a case of a 63-year-old woman who underwent diode laser epilation of the upper eyelids without eye shields; she experienced acute ocular pain during the session and later developed a nuclear cataract and patchy iris atrophy in that eye [48]. In the absence of other risk factors, these findings were attributed to the laser treatment, demonstrating that near-infrared diode laser energy can induce lenticular opacities (lens damage) and iris damage [48]. Likewise, Gulmez et al. reported a case of bilateral anterior uveitis, glaucoma, and permanent iris atrophy with corectopia (pupil distortion) after a cosmetic diode laser application to the eyelids [45]. The mechanism involves the laser’s energy being strongly absorbed by pigmented tissues: the heavily pigmented iris can suffer thermal injury, leading to atrophy and scarring, and inflammation can lead to elevated intraocular pressure (glaucoma) and posterior synechiae (iris sticking to lens) [45]. Alexandrite and diode lasers, which operate in the red/infrared spectrum, are particularly implicated in these iris injuries [45,49]. Proper use of metal corneal shields is imperative for any laser near the eye, and some authors recommend avoiding cosmetic laser procedures on the eyelids entirely due to these risks [45]. In summary, energy-based facial treatments (both IPL and lasers) can cause severe ophthalmologic complications—ranging from corneal injuries and conjunctival burns to anterior uveitis, iris atrophy, corectopia, cataract, and even retinal phototoxicity—if safety protocols are not strictly followed [45]. These events, although rare, underscore the importance of vigilant eye protection and practitioner awareness during non-injectable cosmetic procedures.

## 5. Discussion

This thorough review emphasizes that ophthalmological complications, although rare in aesthetic medicine, vary in presentation and are often difficult to treat. A key point is that prevention is much more effective than treatment for most of these adverse events. Once a filler embolus blocks a retinal artery or a toxin spreads to an unintended muscle, reversing the damage becomes very difficult or impossible. Therefore, understanding risk anatomy, refining injection techniques, and early recognition are essential to reducing the risk of permanent harm. Moreover, with the increasing use of energy-based facial treatments, such as IPL and periocular laser hair removal, additional non-vascular ophthalmologic complications—like uveitis, iris atrophy, and cataract—are being recognized. These injuries, often resulting from a lack of proper eye protection, underscore the importance of caution in all procedures, including non-injectable ones.

The literature consistently shows that central face injections (glabella, nasal dorsum) carry the highest risk for vision-threatening filler complications [4,9]. There is wide agreement that small-gauge cannulas may reduce (but not eliminate) the chance of intravascular injection in these areas by bluntly pushing vessels aside rather than penetrating them. Additionally, practitioners agree on using low injection pressures and volumes, and emphasize the importance of patient feedback during injection (some injectors ask patients about any visual symptoms or pain immediately during the procedure as an early warning). If any signs of intra-arterial injection occur (e.g., blanching or sudden visual changes), the consensus is to stop injection immediately and start first-aid measures (massage, hyaluronidase if HA filler) even as definitive care is being arranged [4].

There is also consensus on management protocols for filler-induced occlusions: despite limited evidence, a practical approach is to administer hyaluronidase (for HA fillers) promptly, use a warm compress and massage to encourage vasodilation and dispersion, begin aspirin (for its anti-platelet effect), and urgently involve ophthalmology and potentially interventional radiology for further treatment [4,7]. The AAO’s 2025 report concluded that although these interventions rarely restore vision, they are generally safe and worth trying in the acute phase. Importantly, none of these should delay transfer to a hospital or stroke center if necessary [7]. The message to ‘call early’ is emphasized—an ophthalmologist or stroke neurologist should be contacted within minutes of a suspected occlusion, as any chance of sight recovery probably depends on opening the artery within 60–90 min. Unfortunately, in practice, many cases have experienced delayed presentation or management (some patients only see an ophthalmologist days later, when nothing can be performed). Moving forward, consensus guidelines recommend that aesthetic providers undergo simulated drills and training so that, in the rare event of an occlusion, they can respond quickly under pressure.

For issues related to botulinum toxin, there is a consensus that these are self-limited problems best managed with conservative measures. Ptosis is typically treated with apraclonidine drops and reassurance for the patient. The literature contains an interesting discussion about whether higher-dilution toxin or certain formulations diffuse more and cause increased ptosis; some authors recommend using onabotulinumtoxinA rather than abobotulinumtoxinA (Dysport, Ipsen Biopharm Ltd., Wrexham, UK) in the forehead, as it may diffuse slightly less and thereby reduce the risk of ptosis [31]. However, the data remain inconclusive. Practitioners agree that precise injection placement—such as staying within the procerus and corrugator muscles and avoiding lateral diffusion—is more important than the specific brand of toxin. Diplopia caused by botulinum toxin is rare enough that no formal guidelines are established, but published case reports and reviews highlight the importance of patient education: patients should avoid rubbing the area after injection and report any double vision immediately [23]. If diplopia occurs, standard recommendations include advising the patient not to drive and providing an eye patch.

Areas of Controversy or Uncertainty: The role of retrobulbar hyaluronidase injections remains debated. Some ophthalmologists strongly advocate for it, citing cases with at least partial vision improvement [27], while others point to instances where it made no difference or possibly caused adverse events, such as optic nerve injury or increased orbital pressure. A 2019 systematic review found no convincing evidence that retrobulbar hyaluronidase alters outcomes [28]. The AAO panel in 2025 took a neutral position: retrobulbar hyaluronidase “remains unproven but may be attempted by an ophthalmologist, recognizing the lack of clear efficacy” [7]. Non-ophthalmologist injectors are generally advised not to attempt retrobulbar injections independently due to risks such as globe injury or hemorrhage, and instead to involve an ophthalmologist. This divergence in practice reflects an evidence gap—since such events are rare, a randomized trial is not feasible, so we depend on case series. In the future, improved fibrinolytic or enzymatic treatments, such as collagenase enzymes for fat emboli or new small-molecule dispersants, may be developed. For now, however, the community has not reached a consensus beyond “try everything reasonable, hope for the best”.

Another area without consensus is the use of hyperbaric oxygen for filler-induced ischemia. Some retina specialists champion HBOT if started within 24–48 h, arguing it can salvage some retinal cells via choroidal oxygenation. Others view it as an adjunct with unproven benefit, especially if started late [27]. Given HBOT’s safety profile, many agree it can be offered if logistically feasible, but not at the expense of proven acute measures. Reports of survivors with vision, like the case of full recovery after HBOT, keep interest alive, though skeptics note that the case may have been a partial occlusion that spontaneously cleared.

It was unexpected to find that even autologous plasma could lead to vision loss following PRP injections [25]. This is still being researched—possible theories include that PRP’s high platelet content can block arteries or that the injected volume caused an embolic hydrostatic event. Since PRP is often injected by cosmetic practitioners who may not be physicians, such as nurses or dentists, there is a need to raise awareness that PRP is not harmless and that the same precautions used for fillers (aspiration, slow injections, avoiding risky areas) should be followed. This could become a growing consensus as more cases are reported.

Limitations of Available Data: Our review is inherently constrained by the quality of published literature. Most included reports are case studies or small series, which are vulnerable to publication bias—unusual or severe cases are more likely to be reported than straightforward ones. Incidence figures are often estimated rather than directly measured, since no large database tracks all cosmetic injections and their complications. Underreporting is probable—for example, a practitioner might treat a mild ptosis in-office without reporting it, or a patient with a small scotoma after filler might never be diagnosed if they do not seek care. Therefore, actual complication rates could be higher than those reported from voluntary surveys [4]. Another limitation is the lack of standardized diagnostic criteria for certain complications; for example, distinguishing between an inflammatory and an infectious nodule often relies on clinical judgment without biopsy, which can lead to misclassification in reports. We also recognize that many management recommendations are based on expert opinion rather than controlled trials. Ethical constraints naturally prevent trials in this area; therefore, clinicians must rely on collective experience and refine their practices as new cases provide valuable insights. This highlights the importance of continued international reporting and collaboration, which have significantly advanced our understanding. Ongoing registries or case collections will further help guide us.

**Future directions** to enhance safety include exploring improved cannula designs and real-time ultrasound guidance for filler injections to reduce the risk of intravascular placement. However, these require additional training and time [50]. Research is also focused on creating alternative reversal agents, such as fibrinolytics for fat emboli, and investigating potential neuroprotective therapies for retinal ischemia.

Patient education remains essential. Individuals should be screened for prior ocular surgeries and instructed to report visual symptoms immediately during or after injection, as delayed recognition has contributed to poor outcomes in some cases. Strengthening collaboration between aesthetic practitioners and ophthalmologists is critical. Ophthalmologists can contribute to training on vascular anatomy and early signs of complications, while aesthetic providers benefit from having emergency referral networks. This multidisciplinary approach ensures faster intervention and more ethical, effective care when complications arise.

## 6. Conclusions

Ophthalmologic complications from aesthetic medicine procedures, although rare, can result in some of the most severe outcomes in all elective medicine. Vision loss, extraocular muscle palsies, and ocular surface damage can result from what are seemingly routine cosmetic treatments. This narrative details the clinical signs and management of these complications, based on a decade of accumulated evidence. The key findings highlight that retinal artery occlusion from filler or fat injections is the most severe complication, often resulting in permanent blindness despite all therapeutic efforts. Botulinum toxin injections can cause notable but temporary issues such as ptosis and diplopia, which typically resolve on their own. Other complications—granulomas, local infections, and dry eye—are generally manageable with medical therapy or minor procedures.

From a preventive standpoint, following best practices and maintaining anatomical safety can reduce (but not eliminate) the risk of serious eye injury. Patients should be fully informed about these risks, even if they are rare, as part of the informed consent process. In the unlikely event of an ophthalmic complication, a quick, coordinated response involving ophthalmology provides the best chance, however small, for a positive outcome.

In conclusion, as the popularity of aesthetic procedures continues to grow, both cosmetic practitioners and eye care professionals must stay vigilant and collaborate closely. Early detection, prompt intervention, and proper referral can reduce the impact of complications. Continued research and reporting are essential to improve management protocols and develop new strategies, such as better filler formulations or antidotes, to enhance patient safety. The experiences of the past decade have turned previously obscure complications into well-understood clinical issues with evolving standards of care. By applying this knowledge in practice, we can ensure that aesthetic improvements are not only attractive but also safe for our patients’ eyes.

## Figures and Tables

**Table 1:** Summary of essential clinical and preventive insights (best practices) regarding ophthalmologic complications associated with aesthetic medicine procedures.

Key Messages
Ophthalmologic complications from aesthetic procedures, though rare, can be severe and irreversible. Prevention is paramount, as effective treatments are often limited.
Retinal artery occlusion (RAO) due to filler embolism is the most devastating complication. Even with prompt treatment, vision prognosis is usually poor. Early recognition and urgent intervention (within minutes) are essential to attempt to mitigate visual damage.
Injections in high-risk facial zones (e.g., glabella, nasal dorsum) have the greatest risk of vision-threatening complications. Use extreme caution if treating these areas (or consider avoiding them for fillers).
Employ safe injection techniques: whenever possible, use microcannulas instead of sharp needles in high-risk areas, inject slowly with minimal pressure (e.g., thumb on plunger), and aspirate before injecting (recognizing that aspiration is not 100% reliable). Small aliquots (<0.1 mL per injection) can reduce the chance of large emboli.
Proper injector training and anatomical knowledge are critical. Many severe complications occurred with injectors lacking specific oculofacial anatomy training. Practitioners should be credentialed and thoroughly trained, especially when operating near the eyes.
Informed consent is essential. Patients should be made aware of even the rare risks (blindness, stroke, etc.) before undergoing procedures.
Be prepared: have a “filler complication kit” ready (e.g., hyaluronidase, aspirin) and an emergency referral pathway to an ophthalmologist on call. At the first sign of visual symptoms, stop injection immediately and initiate emergency management.
Even non-injectable treatments can cause ocular injury. Ensure eye protection (e.g., metal corneal shields) during intense pulsed light (IPL) or laser procedures near the eyes. Improper use of these devices can lead to serious complications like corneal burns, uveitis, iris atrophy, or cataract.
Botulinum toxin complications (ptosis, diplopia) are usually transient and self-limited. Use precise technique (e.g., avoid injecting too close to the orbital rim, correct dosing) to minimize these side effects.
No guaranteed “antidote” exists for many of these complications. Once a filler embolus causes ischemia or a toxin diffuses improperly, reversing the damage is difficult. Thus, prevention remains the main strategy and is far better than any cure.

**Table 2:** Overview of the most relevant ocular complications linked to aesthetic procedures, including estimated frequency, recommended management, and key clinical considerations.

Complication	Estimated Frequency	Management	Relevant Notes
Central Retinal Artery Occlusion (CRAO) [3,4,5,7,9,13,14,15,16,27,28].	1 in 100,000 injections	Urgent: retrobulbar hyaluronidase (if HA), hyperbaric oxygen, ocular massage, antiplatelets. Prognosis is poor.	High visual morbidity. Prompt diagnosis and treatment are critical.
Ischemic Optic Neuropathy (AION/NAION) [7,9,25].	Extremely rare (only isolated cases reported)	Systemic steroids, visual support. Usually irreversible.	May coexist with CRAO. Requires clinical and angiographic assessment.
Diplopia / Extraocular muscle palsy [21,22,23,24,29,30].	0.7–2.2%	Observation, prisms, eye patch. Usually resolves spontaneously.	May be due to botulinum toxin or ischemia from fillers.
Palpebral Ptosis [6,12,31].	2.5%	Topical apraclonidine, observation. Resolution in 2–8 weeks.	Related to toxin diffusion to levator muscle.
Dry Eye/Lagophthalmos [6,31,32].	≈3%	Artificial tears, ointments, night time lid closure. Improves with muscle recovery.	Mainly due to orbicularis oculi paralysis after toxin.
Periocular Skin Necrosis [3,4,5,7,9,25,26].	≈1–3%	Urgent: Hyaluronidase (if HA), antibiotics, hyperbaric oxygen. Prevent exposure-related damage.	Urgent reperfusion is needed to avoid scarring and visual sequelae.
Granulomas/Inflammatory Nodules [33,34].	<1%	Hyaluronidase, intralesional corticosteroids, antibiotics or surgical excision if needed.	Frequent with permanent fillers or biofilm reactions.
Cellulitis / Periocular Infection [33,34,35].	≈6%	Oral or IV antibiotics depending on severity. Drain abscesses if present.	Differential diagnosis of delayed swelling. Requires close monitoring.

## Supplementary Materials

The following supporting information can be downloaded at: https://www.mdpi.com/article/10.3390/jcm14155399/s1, Table S1. Literature Search Strategy and Parameters for Identifying Ophthalmic Complications Related to Non-Surgical Facial Aesthetic Procedures.
