# Supplementary material for: Ophthalmological Complications of Aesthetic Medicine Procedures: A Narrative Review
Source: J Clin Med. 2025 Jul 31;14(15):5399. doi: 10.3390/jcm14155399 (PMC12347381; doi:10.3390/jcm14155399)
Supplement: Supplementary file 1 [file jcm-14-05399-s001.zip › jcm-3755016-supplementary.pdf]

Journal of Clinical Medicine

MDPI

Supplementary Table S1. Literature search strategy and parameters for identifying ophthalmic complications related to non-surgical facial esthetic procedures.

Database	Search Terms Used	Limits Applied
PubMed	("dermal filler" OR "botulinum toxin" OR "fat graft" OR "platelet-rich plasma" OR "PRP" OR "thread lift") OR “Intense Pulsed Light (IPL)” AND ("blindness" OR "retinal artery occlusion" OR "diplopia" OR "ptosis" OR "dry eye" OR "ophthalmoplegia" OR "orbital cellulitis" OR "uveitis" OR "optic neuropathy")	Humans, English, 2015–2025
Embase	('dermal filler'/exp OR 'botulinum toxin'/exp OR 'autologous fat graft'/exp OR 'platelet-rich plasma'/exp) OR “Intense Pulsed Light (IPL)” AND ('ocular complication'/exp OR 'vision loss'/exp OR 'retinal embolism'/exp)	Human studies, 2015–2025
Cochrane Library	dermal filler OR botulinum toxin OR PRP OR Intense Pulsed Light AND vision OR ocular OR blindness	2015–2025
